# Understanding Li-based battery materials via electrochemical impedance spectroscopy

**DOI:** 10.1038/s41467-021-26894-5

**Published:** 2021-11-11

**Authors:** Miran Gaberšček

**Affiliations:** 1 Department of Materials Chemistry, National Institute of Chemistry, Hajdrihova 19, 1000 Ljubljana, Slovenia; 2 Faculty of Chemistry and Chemical Technology University of Ljubljana, Večna pot 113, 1000 Ljubljana, Slovenia

**Keywords:** Electrochemistry, Batteries, Energy, Batteries, Materials for energy and catalysis

## Abstract

Lithium-based batteries are a class of electrochemical energy storage devices where the potentiality of electrochemical impedance spectroscopy (EIS) for understanding the battery charge storage mechanisms is still to be fully exploited. Generally considered as an ancillary technique, the application of EIS should be promoted focusing on improved experimental design of experiments and advanced data analysis using physics-based models.

## Abstract (web summary)

Electrochemical impedance spectroscopy is a key technique for understanding Li-based battery processes. Here, the authors discuss the current state of the art, advantages and challenges of this technique, also giving an outlook for future developments.

## Electrochemical impedance spectroscopy—a powerful in situ electrochemical technique

Electrochemical impedance spectroscopy (EIS) is a powerful technique for investigating processes occurring in electrochemical systems. Generally, such processes involve the dynamics of bound or mobile charge in the bulk or interfacial regions of any liquid or solid material: ionic, semiconducting, mixed electronic-ionic and even insulators (dielectrics)^1^. The main strength of EIS is its ability to effectively deconvolute complex electrochemical processes into a series of basic processes based on the different relaxation times. However, the system must remain in a stationary state throughout the EIS measurement. Both features can be achieved using a small-amplitude potential or current periodic perturbation to excite the electrochemical system at different frequencies. By measuring the response (current or potential) of the system to this perturbation, the corresponding transfer function, that is the impedance of the system, can be calculated^2^. In the ideal case, the impedance spectrum contains a separate feature for each elementary process that constitute the overall electrochemical mechanism.

Already a basic EIS measurement of a typical electrochemical energy storage cell, in which the whole system between both cell’s electrodes is probed, may produce a spectrum in which the reaction(s) that occur on the positive and negative electrode are observed as (well) separated features (e.g. semi-circles in the complex impedance plots). Additionally, the migration of ions across the liquid electrolyte contained in a separator is observed as a high-frequency intercept along the x-axis of the complex impedance plot (also called the Nyquist plot). More elaborated EIS studies of a given cell may help identify other basic processes such as (i) formation of surface films on electrode materials, (ii) poor inter-phase contacting and (iii) depletion of carries in either active phase or electrolyte. Below we briefly discuss the advantages and drawbacks of this in situ technique taking into account the best-known modern electrochemical energy storage system: the lithium-ion battery.

## Electrochemical impedance spectroscopy of lithium-ion batteries

Lithium-ion batteries (LIBs) have been intensely and continuously researched since the 1980s. As a result, the main electrochemical processes occurring in these devices have been successfully identified. However, the detailed nature of specific mechanisms, such as the effect of charge/discharge rate or prolonged cell cycling on the energy and power storage performance, is still not sufficiently understood. These aspects are crucial and strongly affect, e.g. the lifetime and cost of LiBs and must be implemented to improve the overall quality of a LiB device^3^. In this regard, EIS could be considered as a useful technique that may generate insights to help solve the not yet addressed LiB issues.

A literature survey using databases such as Scopus or Web of Science reveals that EIS is not frequently used in lithium-based battery studies (i.e. only about 6000 research articles out of 115,000 covering LiBs disclose EIS measurements and analyses). Furthermore, the large majority of those articles include EIS as a supporting technique, i.e. an additional technique that mainly confirms the trends already found via conventional electrochemical measurements (e.g. constant current potentiometry, cyclic voltammetry, cycle testing, etc.). To some extent, such rather trivial application of EIS in the field of batteries can be well understood: modern EIS devices allow for fast data acquisition and, at the same time, their interpretation is facilitated using automated algorithms, most frequently in terms of equivalent circuit analysis^2^. In short, performing and interpreting a basic EIS measurement is relatively straightforward; thus, including such data in a research article is rather convenient.

By contrast, if the scientific research community wishes to exploit the potential of this powerful technique fully, considerable efforts are needed in terms of measurements optimisation and data interpretation. In continuation, we present several examples of advanced approaches to EIS measurements of LiB systems and state-of-the-art modelling tools for in-depth interpretation of measured data.

## Measurements

The electrochemical performance of a LiB (e.g. maximum capacity, rate capability, cycle efficiency and stability) is usually evaluated using a full cell consisting of two different positive and negative electrodes. Most frequently, the same two-electrode full cells are also used for EIS measurements. However, full cells contain many elementary processes stemming from each of the electrodes, which are extremely difficult to deconvolute from a single measured spectrum properly, despite the inherent resolution of EIS (i.e. the ability to resolve complex electrochemical processes into individual steps)^4^. Thus, if we are interested in mechanisms rather than overall cell performance, specific cell configurations and geometries must be considered. An option that has proved quite helpful is the symmetric cell configuration that consists of two identical electrodes^5^ which could identify a smaller number of elementary processes, halved compared to a full asymmetric cell (i.e. where the electrode tested are not identical). Symmetric cells can be assembled from pristine (e.g. not electrochemically tested) or postmortem (i.e. electrochemically tested) electrodes, the latter harvested from disassembled full cells. Another option that also halves the number of observed elementary steps is using three-electrode cells, which additionally include a separate reference electrode. However, the latter configuration only probes the processes on the working (selected) electrode. Moreover, the correct positioning of the reference electrode and its chemical nature are crucial for acquiring reliable experimental data^6^.

**Fig. 1:**
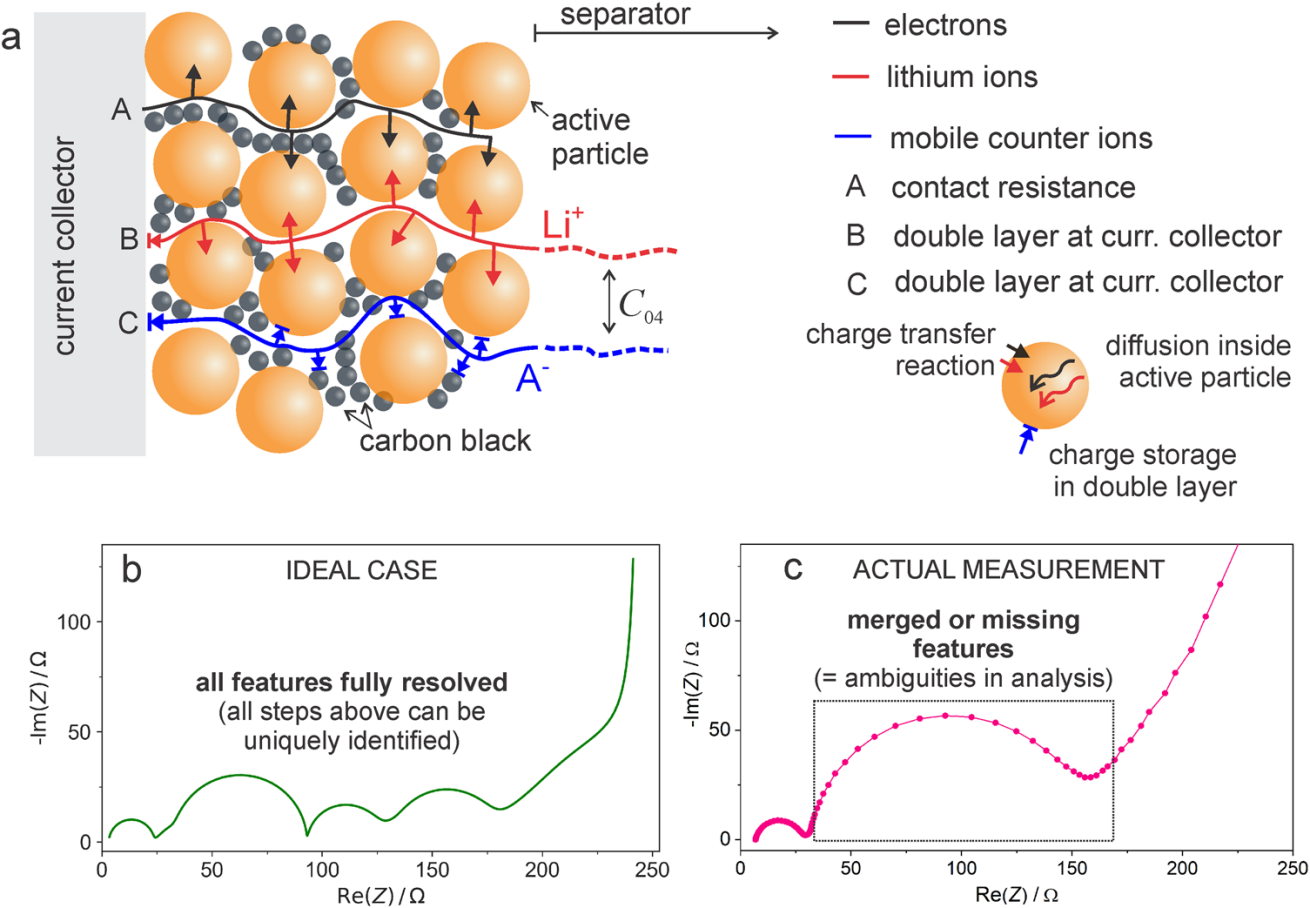
Typical processes in a lithium-ion battery electrode and their identification using electrochemical impedance spectroscopy measurements. The basic scheme showing the electrode structure in panel a was taken from ref. ^7^. **a** Schematics showing the movement of electrons and mobile ions in a typical Li-ion insertion positive electrode. **b** Theoretical impedance response for an ideal case where each individual step shown in **a** can be seen as a separate feature. **c** Example of a practical EIS measurement where many of the predicted features are not seen due to overlap of time constants, very small values of impedance values for certain steps or other measurement artefacts. Most of the missing features can be retrieved using dedicated electrochemical experiments, as explained in the main text.

Even when using symmetric or three-electrode cells, the number of elementary processes taking place in the cell may remain high and typically involves (i) transfer of electrons from the current collector to the electrode composite, (ii) electron conduction/migration across the composite electrode thickness, (iii) ion migration across the electrode thickness, (iv) electrochemical insertion of ion and electron into the active storage particles, (v) double-layer charging at solid/liquid interfaces, (vi) coupled diffusion of active and non-active ions in porous electrode composite, (vii) coupled diffusion of ion and electron inside the active storage particles and (viii) migration and diffusion of ions in separator^7^. In fact, in the ideal case, EIS is able to detect separately more or less all of these processes as individual features in a single measured spectrum (Fig. 1). To be precise: in the ideal case, the number of measured features is only one less than the number of individual processes^4,7^ which shows the capability of EIS to split the complex processes into their elementary steps. The problem is that many of these individual features overlap in realistic measurements, and it is rather challenging to decouple them unambiguously^4,7^. This aspect is crucial and must be carefully considered to exploit EIS in the battery research field fully.

During the past decade, scientists have proposed several experimental approaches to effectively decouple the merged parts of a LiB impedance spectrum into individual features. These approaches can be divided into several categories. The first considers the systematic variation of the cell components and analysis of the corresponding changes in measured EIS spectra. For example, a change in the electrolyte concentration will only affect the parts of the spectrum where migration and diffusion in the electrolyte phase respond to the excitation signal but leave the other parts unchanged, thus facilitating the identification and analysis of the battery component under investigation^4^. Similarly, modification of the electrode thickness, size of the active material particles, number and/or thickness of separator(s) and the chemistry of mobile ionic species, may selectively affect the measured spectrum and greatly facilitate the identification and analysis of individual mechanistic steps.

Another approach that may crucially contribute to a correct and quantitative interpretation of impedance spectra relies on combining EIS data with data obtained from complementary techniques. Namely, the theoretical models that are used for the interpretation of EIS spectra involve many parameters related to the microstructure, morphology or chemical composition of cell components such as particle size^8^, electrode thickness^9^, porosity and tortuosity^10^, nature and concentration of mobile and immobile species^11^. Thus, any technique that can provide such data can significantly contribute to the quality of analysis of the EIS spectra. A range of ex- or in-situ complementary techniques such as visual inspection of the specimen, a range of microscopic techniques combined with local chemical analysis, diffraction techniques, infra-red and nuclear magnetic spectroscopies, chromatographic techniques and others have recently been used to upgrade the electrochemical data^12^.

To better resolve the features in EIS spectra, researchers occasionally also report the use of so-called dynamic EIS^13^ where the small alternating current (a.c.) perturbation signal is superimposed on the direct current (d.c.) bias that mimics the charge or discharge conditions of a Li-ion cell. However, unlike in other fields, such as corrosion or fuel cells, the use of a d.c. base signal can be highly problematic in the case of insertion electrode active materials. This is because a d.c. signal changes the stoichiometry of the active material, which in turn affects the spectrum and thus violates the condition of stationarity of the system during the EIS measurement^1,2^. Therefore, special approaches are needed to implement the effects of d.c. bias into EIS measurement of insertion-based battery active materials in a consistent way^13^.

## Analysis of data

Analysis of measured impedance data can be carried out on several well-established levels. The first is known as the equivalent circuit analysis and largely dominates the field of batteries. This analysis relies on the fact that, in terms of impedance, the most frequent processes such as migration of charge through a phase and accumulation of charge at a phase boundary exhibit the same response as ordinary macroscopic electric resistors and capacitors, respectively. Similarly, diffusion of charge can also be presented using a particular electric element, e.g. the Warburg impedance. Combining such electric elements, one can relatively easily and accurately fit the basic shape of most of the measured spectra. However, many different combinations of elements can give similar (or even identical) spectra, so the use of equivalent circuit analysis is only recommended in cases that have been previously proven correct using physics-based modelling presented in continuation. For example, the Randles circuit is the best-known example of an equivalent circuit with a clear physical background^1^.

Unlike equivalent circuit analysis, physics-based approaches to the analysis of impedance spectra rely on applying general physical laws that describe the transport of mass and charge and electrochemical reactions in solid or liquid phases and at their interfaces. The most direct way of such treatment is finding analytical solutions^14^ of the governing equations that describe the assumed transport-reaction mechanism in a given cell of interest and then comparing (fitting) the calculated spectra to the measured EIS responses. In cases where analytical solutions are not known, of course, numerical methods can be used. The fast development of simulation tools has recently allowed for the creation of in silico 3D electrode structures where the impedance is analyzed using appropriate software^11^. A third physics-based approach that has a long tradition in the field of EIS relies on direct transcription of governing equations into electric circuit elements^4,7^. This approach results in large structures containing thousands of physically well-defined electric elements, generally known as transmission lines. Unlike conventional equivalent circuits, which can only describe macroscopically homogeneous systems, the elements of a transmission line can accurately describe the many local processes/steps occurring in an electrochemical system. The benefit of transmission line modelling compared to other physics-based approaches is the direct visualisation of the complex processes taking place in a Li-ion cell^1,4,14^. This model is also much faster than other numerical procedures^4^.

## Summary and outlooks

EIS is a powerful electrochemical technique that can resolve complex processes into their fundamental steps. However, in the field of batteries, the great potential of this technique has not been fully exploited yet. In order to do so, researchers are encouraged to design special sets of impedance-centred experiments using dedicated electrodes or even custom-built cells at a range of variable but well-controlled conditions (e.g. varying the concentration and chemical nature of active ions, the electrode thickness, porosity, tortuosity, the size and distribution of active particles, the nature and contents of additives). Such impedance-oriented experimental designs may provide radically new insights into battery mechanisms if such well-controlled impedance studies are combined with complementary (in operando mode when possible) techniques that give information about the macrostructure, microstructure and composition of the system under investigation.
